# Identification and characterization of an operon, *msaABCR,* that controls virulence and biofilm development in *Staphylococcus aureus*

**DOI:** 10.1186/1471-2180-14-154

**Published:** 2014-06-11

**Authors:** Gyan S Sahukhal, Mohamed O Elasri

**Affiliations:** 1 Department of Biological Sciences, The University of Southern Mississippi, Hattiesburg, Mississippi 39406-0001, USA

**Keywords:** *Staphylococcus aureus*, *msaABCR* operon, Biofilm, Virulence factor

## Abstract

**Background:**

Community-acquired, methicillin-resistant *Staphylococcus aureus* strains often cause localized infections in immunocompromised hosts, but some strains show enhanced virulence leading to severe infections even among healthy individuals with no predisposing risk factors. The genetic basis for this enhanced virulence has yet to be determined. *S. aureus* possesses a wide variety of virulence factors, the expression of which is carefully coordinated by a variety of regulators. Several virulence regulators have been well characterized, but others have yet to be thoroughly investigated. Previously, we identified the *msa* gene as a regulator of several virulence genes, biofilm development, and antibiotic resistance. We also found evidence of the involvement of upstream genes in *msa* function.

**Results:**

To investigate the mechanism of regulation of the *msa* gene (renamed *msaC*), we examined the upstream genes whose expression was affected by its deletion. We showed that *msaC* is part of a newly defined four-gene operon (*msaABCR*), in which *msaC* is a non-protein-coding RNA that is essential for the function of the operon. Furthermore, we found that an antisense RNA (*msaR*) is complementary to the 5′ end of the *msaB* gene and is expressed in a growth phase-dependent manner suggesting that it is involved in regulation of the operon.

**Conclusion:**

These findings allow us to define a new operon that regulates fundamental phenotypes in *S. aureus* such as biofilm development and virulence. Characterization of the *msaABCR* operon will allow us to investigate the mechanism of function of this operon and the role of the individual genes in regulation and interaction with its targets. This study identifies a new element in the complex regulatory circuits in *S. aureus*, and our findings may be therapeutically relevant.

## Background

*Staphylococcus aureus* is an important human pathogen that causes a wide range of infections, from superficial to systemic [1,2]. The ability of *S. aureus* to infect a variety of tissues is due to its expression of a wide variety of virulence factors. These factors are categorized as surface-associated proteins, secreted proteases, toxins, or immune modulators [3]. Expression of virulence factors in *S. aureus* is carefully coordinated by a variety of regulators that include trans-acting global regulators, alternative sigma factors, and small non-coding RNAs [3-5]. Indeed, the *S. aureus* genome has 124 putative transcriptional regulators [6]. Understanding virulence regulation during growth under different environmental conditions (e.g., biofilm development) is imperative for the effective prevention and treatment of *S. aureus* infections. To date, several global regulators have been identified, which include the *agr* operon, the *sarA* gene family, the *saePQRS* operon, and the genes *arlRS, lytSR, srrAb, hssRS, vraSR*, and *graSR*[7-16]*.* Several other regulators have also been identified, though they are not as well characterized (e.g., *htrA, ccpA, msrR,* and *svrR*) [17-20]*.*

The *msa* gene, henceforth referred to as *msaC*, was initially identified as a regulator of *sarA, agr,* and several virulence factors [21]. We previously reported that *msaC* is also involved in biofilm development [22]. Indeed, we showed that deletion of the *msaC* gene resulted in a significant defect in accumulation of biofilm but did not affect the initial adherence to surfaces [22]. However, it was not clear if *msaC* regulated virulence genes directly or via its effect on global regulators. For instance, we found that in the *msaC* deletion mutant, *sarA* expression was reduced during biofilm growth. Since *sarA* has been shown to be essential for biofilm development in several strains [23], it is not clear if the *msaC* defect is due to the reduction in *sarA* or other factors. Sequence analysis of the *msaC* gene showed that it is conserved among *S. aureus* strains and suggested that it encodes a putative membrane protein [21]. We have been unable to express this putative protein, and therefore the mechanism of regulation by *msaC* remains to be determined. The pleiotropic phenotypes in the *msaC* mutant suggested that they were mediated by the global regulators *sarA, agr,* and *sigB*. However, deletion of the *msaC* gene leads to a decrease in the expression of the upstream gene (*cspA*), a gene that is not regulated by *sarA*, *agr*, or *sigB*[21,24]. These findings led us to hypothesize that *msaC* regulated some genes (e.g., *cspA*) directly. Here, we examined the relationship between *msaC* and *cspA* and showed that the *msaC* gene is part of a four-gene operon.

## Results

### *msaC* is a member of a four-gene operon

**Figure 1:**
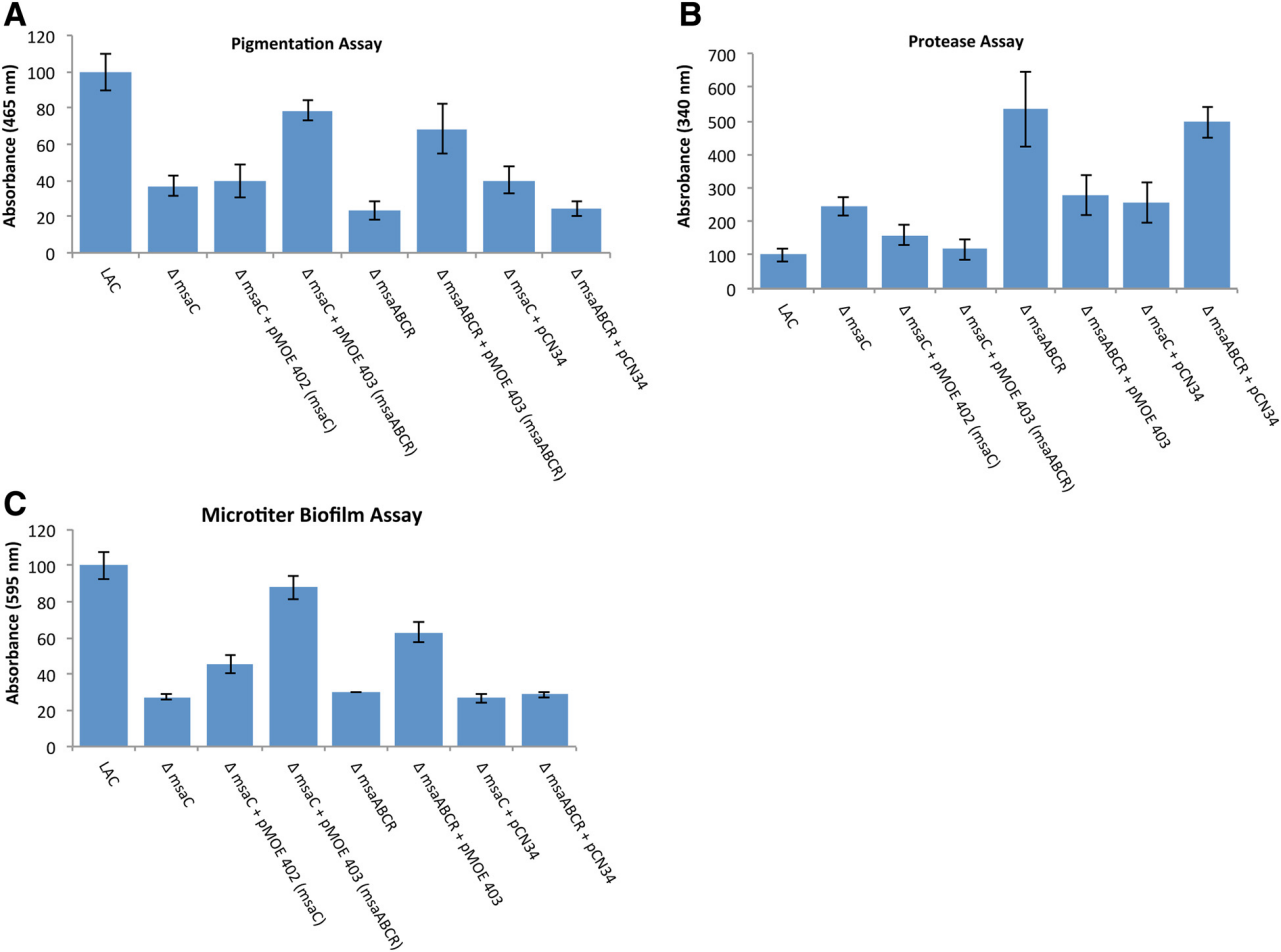
**Phenotypic analysis of the *msaC *and *msaABCR *deletion mutants and the complemented strains.** Comparison of the *msaC* and *msaABCR* deletion mutant phenotypes relative to the complemented mutants and the wild type USA300 LAC strain. The following phenotypic assays were performed: **(A)** pigmentation; **(B)** production of extracellular proteases; and **(C)** biofilm formation. Values are shown as the percent activity relative to the wild type strain USA300 LAC, which was set as 100%. Values represent the average of three independent assays which were done in triplicate. Results are reported as the mean ± S.E.

Previously, we showed that *msaC* regulates the expression of *sarA*, and is essential for biofilm formation and protease production [21,22]. One of the early observations about *msaC* was that expression of the upstream gene (SAUSA300*_*1295) was significantly reduced (two-fold) by deletion of *msaC*[21]. In addition, some assays involving expression of upstream genes did not show full complementation when we introduced the *msaC* gene alone into the *msaC* mutant. To investigate this further, we deleted the *msaC* gene from the *S. aureus* community-acquired strain, USA300 LAC, and complemented the mutant with a region of the chromosome that included upstream and downstream genes relative to *msaC*. Introduction of three open reading frames (ORFs) (encoding SAUSA300_1296, SAUSA300_1295, and *msaC*) resulted in full complementation to restore the wild type phenotype in biofilm formation, host protein binding assays, protease production, and expression of *sarA*. These findings suggested that the *msaC* gene is part of an operon that included at least three genes. To test this hypothesis, we deleted all three genes (encoding SAUSA300_1296, encoding SAUSA300_1295, and *msaC*) in the USA300 LAC strain and compared the phenotype to the *msaC* single-deletion mutant. Several phenotypic variables were examined in the two mutants and similar phenotypes were observed. Both mutants showed reduced pigmentation and biofilm production and increased protease activity relative to wild type (Figure 1). Both mutants also showed a downregulation in the expression of key global regulators, *sarA*, *agrA*, and *sigB* (Table 1). The phenotype of the three-ORF-deletion mutant was similar to the *msaC* mutant, with one exception being that the three-ORF-deletion mutant produced significantly more proteases than the *msaC* mutant (Figure 1B). Furthermore, complementation studies in the *msaC* deletion mutant confirmed that all three ORFs were required for restoration of the wild type phenotype (Figure 1, Table 1). These findings suggested that the three genes are functionally related and form an operon.

**Table 1:** **Relative expression of global regulators ( *sarA *, *agr*, *sigB*) in the *msaC *** **and *msaABCR *** **deletion mutants**

**Strain**	Fold change*		
	***sarA***	***agrA***	***sigB***
LAC	1	1	1
∆ *msaC*	-3.03	-5.72	-2.37
∆ *msaC* + pMOE402 (*msaC*)	-3.45	-6.74	-2.23
∆ *msaC* + pMOE403 (*msaABCR*)	-1.74	1.74	-1.35
∆ *msaABCR*	-3.59	-6.7	-2.07
∆ *msaABCR* + pMOE403 (*msaABCR*)	-1.39	-1.56	1.01

*Fold change in the transcription level in the various strains relative to wild type (USA300 LAC). Values represent the mean ratio for at least three independent experiments.

**Figure 2:**
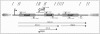
**Map of the *msaABCR *operon.** Rapid amplification of cDNA ends (RACE) and Northern blot analyses were used to determine the length and ends (5′ and 3′) of the RNA transcripts produced by the *msaABCR* operon. Long thin arrows represent the various RNAs identified in this study, the direction and length in nucleotides (nt) is also indicated. Short thick arrows indicate the positions and directions of the gene-specific primers used in the separate reactions. Reporter gene fusion studies showed the presence of only one active promoter (P) in this operon.

**Figure 3:**
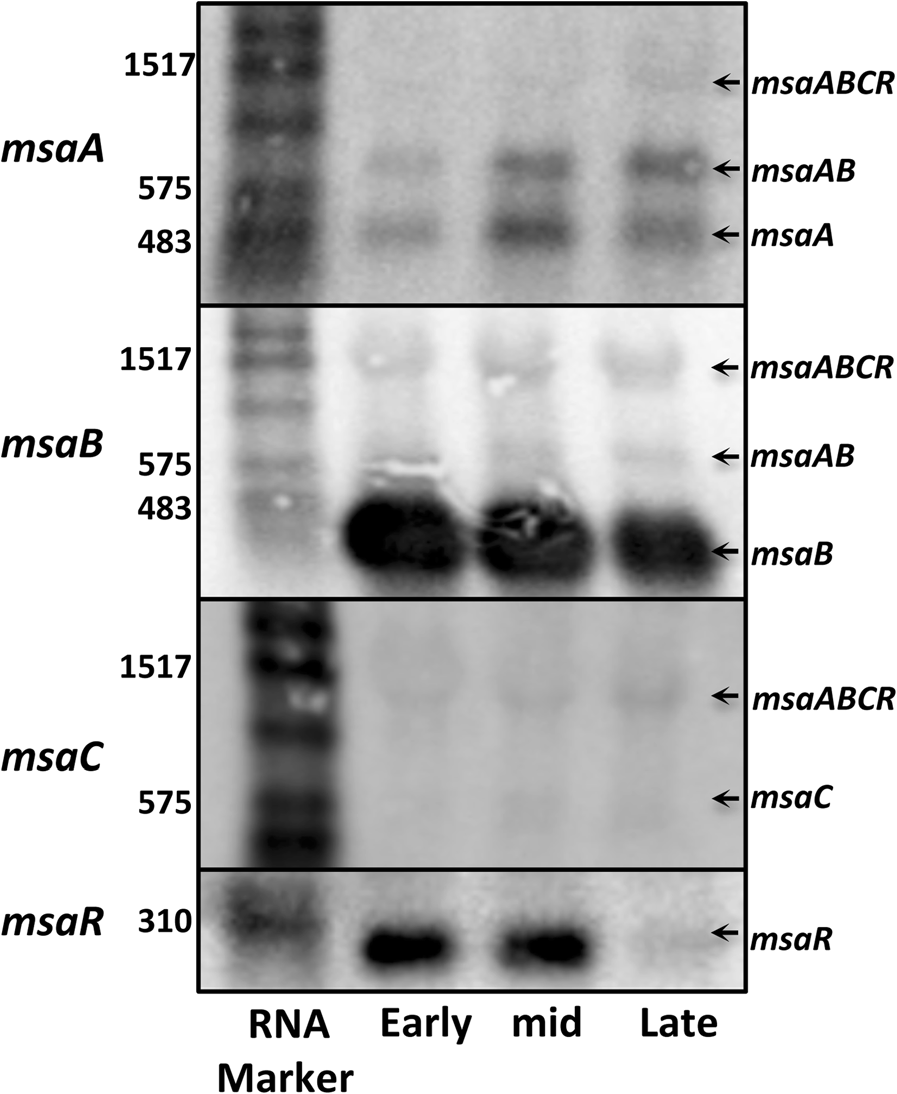
**Transcription of the *msaABCR *operon.** Northern blot analyses were performed to detect transcripts produced by the *msaABCR* operon at three growth phases (early exponential, mid exponential, and late exponential). Gene specific riboprobes were used and are indicated on the left. The identity of the transcripts detected is indicated on the right. Size marker (nt) is shown on the left.

These findings led us to analyze the transcript produced by these three ORFs using rapid amplification of cDNA ends (RACE) and Northern blot analyses. We used the SMARTer™ RACE cDNA Amplification kit (Clontech) to analyze the mRNA transcripts harvested from the USA300 LAC strain. We used several gene-specific primers to find the 5′ and 3′ ends of all transcripts produced by this operon. The RACE reaction products were separated by agarose gel electrophoresis, and the different DNA bands observed were gel purified and sequenced. The results from RACE analysis (5′ and 3′) showed the production of a 1541-nt RNA whose 5′ end was located 35 nt upstream of the gene encoding SAUSA300_1296 (renamed *msaA*), while its 3′ end was located 230 nt downstream of the gene encoding SAUSA300_1294 (*msaC*). The ends of each transcript were confirmed using at least two different primers and three independent reactions for each primer (Figure 2). Results of the RACE analysis were confirmed by Northern blot analysis using gene-specific probes (Figure 3).

The findings from RACE and Northern analyses confirmed that *msaC* is part of a three-gene operon that comprises a gene that encodes for a hypothetical protein (SAUSA300_1296), a gene that is similar to the *E. coli* cold-shock gene (*cspA*, encoding SAUSA300_1295), and the *msaC* gene (encoding SAUSA300_1294) (Figures 2 and 3). This indicated that all three genes were functionally related and involved in biofilm development, protease production, and regulation of the *sarA*, *agr*, and *sigB* genes. We named this operon *msaABCR*, where *msaA* encodes SAUSA300_1296, *msaB* encodes SAUSA300_1295, *msaC* encodes SAUSA_1294 (originally named *msa*), and *msaR* codes for an anti-sense RNA (see below).

Another interesting finding from RACE and Northern blot analyses was that in addition to the large transcript, there were three sub-transcripts corresponding to *msaAB*, *msaB*, and *msaC* (Figures 2 and 3). Additionally, Northern blot analysis using an *msaA*-specific riboprobe showed the presence of a transcript that corresponded to *msaA* alone, however, we were not able to confirm the ends of this transcript by RACE (Figure 3). Northern blot analysis also revealed that *msaB* was the most abundant transcript produced from this operon, whereas the large *msaABC* transcript and the *msaC* transcript were present at a much lower level (Figure 3). The expression level of these transcripts was further confirmed by real-time quantitative PCR (RT-qPCR) (Additional file 1: Figure S1). These results suggested that the large transcript undergoes post-transcriptional processing to produce the final *msaB* transcript. However, the mechanism of production of the abundant *msaB* transcript requires further studies.

### The *msaABCR* operon contains an anti-sense RNA (*msaR*)

We used a sense riboprobe that hybridizes to the *msaB* region and performed Northern blot analysis. The probe detected an anti-sense RNA that partially overlaps with the *msaB* transcript. We performed RACE analysis to identify the ends of the anti-sense RNA and found that it is 133 nt in length and is complementary to 112 nt of the 5′ UTR region of *msaB* and 18 nt of the *msaB* ORF region (Figure 2). Our results are supported by the identification of this anti-sense RNA in a screen of endoribonuclease III targets in the *S. aureus* strain RN6390 [25]. Lioliou *et al*. [25] found that this 133-nt RNA is involved in regulating the stability of the *cspA* (*msaB*) mRNA. To investigate this further, we measured the expression level of this anti-sense transcript at three growth phases and found that it was produced at early-exponential and mid-exponential phases but it was absent at late-exponential phase (Figure 3). The absence of the anti-sense transcript in the late-exponential growth phase may be due to lack of production or degradation. This differential expression may play an important role in the activity of the operon and suggests that regulation of the operon by *msaR* might be growth-phase dependent.

### Detectable promoter activity within the operon is limited to *msaA*

**Figure 4:**
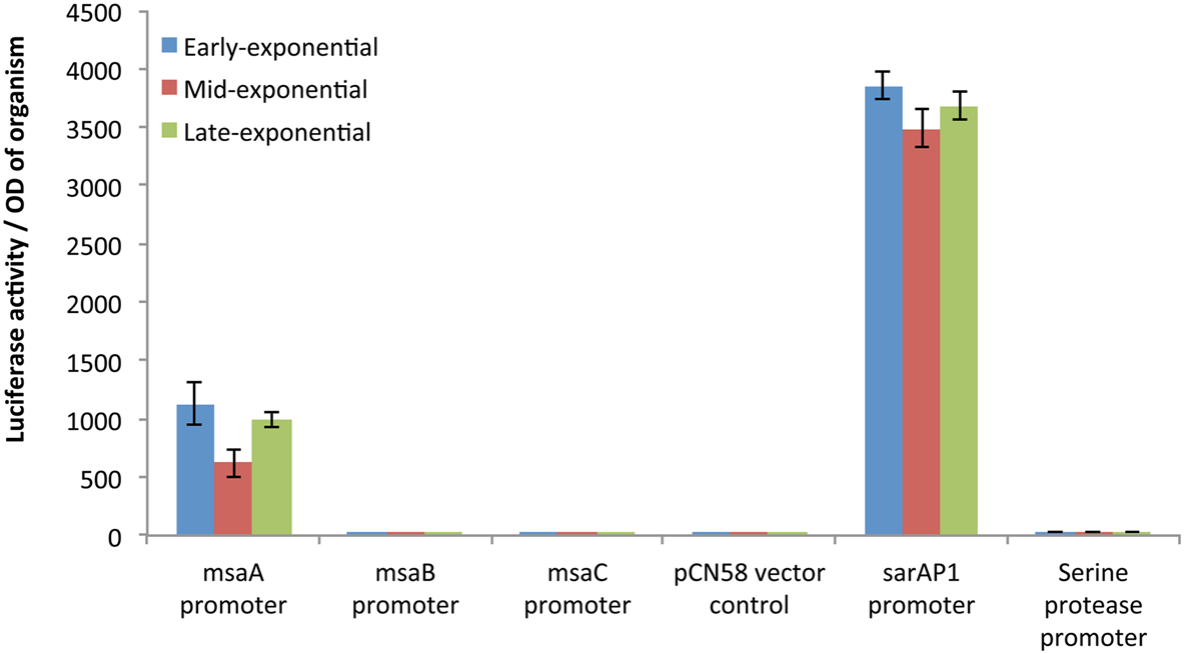
**Promoter activity of *msaA*, *msaB*, and *msaC *at three growth phases.** Putative promoters were fused to promoter-less *luxAB* genes and introduced into the wild type strain USA300 LAC. Luciferase activity was measured at three growth phases (early, mid and late exponential). The vector pCN58 containing *luxAB* without a promoter was used as a negative control. *sarAP1* and serine protease promoters were used as positive controls. Results represent means of three independent experiments where each measurement was done in triplicates. Standard error bars are shown.

RACE and Northern blot analyses showed that, in addition to the large transcript (*msaABC*), the operon produces four sub-transcripts (*msaAB, msaB, msaC*, and *msaR*). These transcripts might be produced from individual promoters or via processing of the large transcript. To test this, we fused the putative promoters from all of the genes in the operon to a promoterless luciferase gene, *luxAB*. We used two promoter-prediction software packages, PePPER (prediction of prokaryote promoter elements and regulon) [26] and Virtual footprint (promoter analysis version 3.0) [27], to select the putative promoters. The promoters included 250 to 300 bp of sequence upstream of each ORF. Selection of the putative promoter regions was also based on the results of RACE analysis by considering every detected 5′ end as a possible transcription start point. We introduced the fusions into the wild type strain and assayed for light production in three growth phases (early-exponential, mid-exponential, and late-exponential). We found that under these growth conditions, the *msaA* promoter was active in all three-growth phases (Figure 4). However, the putative promoters for *msaB* and *msaC* did not show any detectable light production (Figure 4). Compared to controls, the *msaA* promoter shows higher activity than the serine protease promoter but lower than the *sarA*P1 promoter (Figure 4). These findings further confirmed that a single promoter drives expression of the genes in the *msaABCR* operon and that the sub-transcripts probably arise from processing of the main transcript. However, we cannot completely rule out the function of the other putative promoters, which may only be active under growth conditions that we have not yet tested.

To further examine the potential role of the *msaA* promoter in the regulation of expression of the operon, we introduced *msaA–luxAB* fusions into the two deletion mutants, the *msaABCR* operon, and *msaC*. We found that the promoter activity was significantly increased (>2 fold) in both mutants, indicating a negative auto-regulation mechanism that controls the expression of the operon (Additional file 2: Figure S2). Interestingly, this increase in expression was observed in three phases of planktonic growth as well as in biofilms.

### *msaC* is a non-protein-coding RNA

**Figure 5:**
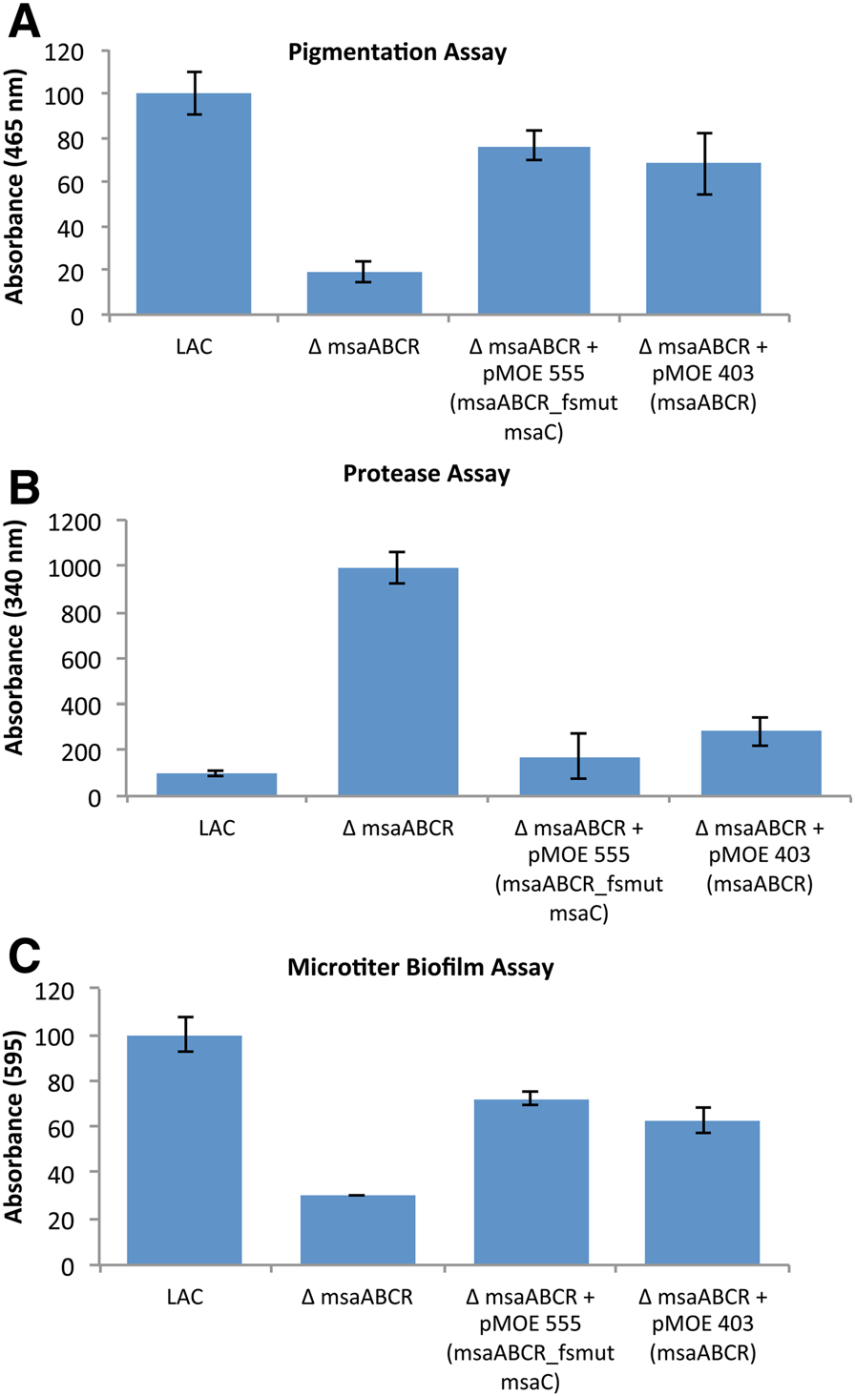
**Phenotypic analysis of the *msaABCR *deletion mutant complemented with the frameshift *msaC *mutation.** The *msaABCR* operon construct containing the frameshift *msaC* mutant (*msaABCR*_fsmut *msaC*) was introduced in the *msaABCR* operon deletion mutant. The following phenotypic assays were performed: **(A)** pigmentation; **(B)** production of extracellular proteases; and **(C)** biofilm formation. Values are shown as the percent activity relative to the wild type strain USA300 LAC, which was set as 100%. Values represent the average of three independent assays, which were done in triplicate. Results are reported as the mean ± S.E.

Studies on the *msaC* gene were initiated by the discovery of its role in the expression of *sarA* and several virulence factors. In this study, we showed that *msaC* is part of a four-gene operon, *msaABCR*, and that its deletion leads to a phenotype that is equivalent to deletion of the whole operon. Lack of similarity of the predicted protein sequence encoded by *msaC* with protein databases and our failure to express the protein using several approaches led us to hypothesize that *msaC* is a non-coding RNA. We constructed a mutant in the *msaABCR* operon that contained a frameshift mutation in the *msaC* ORF that did not significantly change the predicted secondary structure of the RNA (Additional file 3: Figure S3.A). This frame shift mutation led to the introduction of several nonsense mutations in the putative *msaC* ORF (Additional file 3: Figure S3.D). We then introduced the mutated operon (*msaABCR*_fsmut *msaC*) into the *msaABCR* deletion mutant. We compared the phenotype of this strain to the wild type strain and to the *msaABCR* deletion mutant complemented with wild type *msaABCR*. We found that the frameshift mutant operon (*msaABCR*_fsmut *msaC*) complemented the deletion mutant in pigmentation, protease production, biofilm development, and expression of three global regulators *sarA*, *agrA, and sigB* (Figure 5, Table 2). The level of complementation between the *msaABCR* operon and *msaABCR*-fsmut-*msaC* did not show a statistically significant difference (Figure 5 and Table 2). These results suggested that the *msaC* gene does not encode a protein. The absence of the MsaC protein from all current proteomic studies in *S. aureus* further supported the conclusion that *msaC* produces a non-protein-coding RNA that is required for the expression of the *msaABCR* operon [28,29].

**Table 2:** **Relative expression of global regulators in *msaABCR *** **deletion mutant complemented with the frameshift *msaC *** **mutation**

**Strain**	Fold change*			
	***sarA***	***agrA***	***sigB***	***msaC***
LAC	1	1	1	1
∆ *msaABCR*	-3.59	-6.7	-2.07	0
∆ *msaABCR* + pMOE555 (*msaABCR_*fsmut *msaC*)	-1.25	-1.04	-1.08	17.95
∆ *msaABCR* + pMOE403 (*msaABCR*)	-1.39	-1.56	1.01	16.37

*Fold change in the transcription level in the various strains relative to the wild type (USA300 LAC).

Values represent the mean ratio for at least three independent experiments.

## Discussion

Mutation of the *msaC* gene was shown to have a pleiotropic effect on the expression of a variety of genes that are involved in virulence, biofilm development, and pigmentation [21,22]. In this study, we showed that the *msaC* gene is part of a four-gene operon that includes two putative RNA regulators (*msaC* and *msaR*). We also showed that *msaABCR* interacts with three global regulators *sarA*, *agr*, and *sigB*. The location of the *msaC* gene in an operon is a significant finding because it has allowed the identification of other genes that are functionally related, and it will facilitate the study of the mechanism of regulation by this operon.

Sequence analysis of the genes in the *msaABCR* operon showed that the putative protein produced by *msaA* is a conserved hypothetical protein in *S. aureus*. We used the I-Tasser program to predict protein structure and function of the putative MsaA protein and found that it has strong similarity to the twin-arginine signal-binding protein [30,31]. In many bacterial systems, twin-arginine transport is used for translocation of folded proteins across the cytoplasmic membrane [32]. In *S. aureus*, however, no such system has been described in detail. Analysis of the predicted MsaA structure also revealed that it is likely located in the cytoplasm and that it might be involved in the regulation of small GTPase-mediated signal transduction [30,31]. The contribution of the putative MsaA protein to the function of the operon remains unclear.

The *msaB* gene encodes a 66-amino acid polypeptide that showed homology with cold-shock proteins of *E. coli* (CspA, 60%) and *Bacillus subtilis* (CspB, 76%). Based on sequence homology, *S. aureus* produces three proteins (CspA, CspB, and CspC) that may be associated with cold-shock stress [33-35]. However, Anderson *et al*. [36] showed that only CspB responds to cold shock in *S. aureus*. This was confirmed by proteomic studies that showed increased expression of CspB under cold shock while CspA was not differentially expressed. Studies have also shown that the *cspA* transcript is more abundant than *cspB* and *cspC* under normal growth conditions at 37°C, while the *cspB* transcript predominates at 15°C [36]. In addition, Katzif *et al*. [33,34] showed that *cspA* is important for the cationic antimicrobial peptide of human lysosomal cathepsin G and regulates pigmentation in *S. aureus* through a *sigB*-dependent mechanism [33,34]. This indicates that CspA has biological functions other than the cold-shock response. Our findings support this conclusion as we show that MsaB (CspA) acts as a regulator of several genes that are involved in protease production, virulence, and biofilm development. At this point, it is not clear how this protein interacts with other factors, but identification of the *msaABCR* operon will allow us to investigate this mechanism and further characterize this new regulator of virulence.

In *E. coli*, the *cspA* mRNA is a thermosensor that modulates translation of the cold-shock protein (CspA). The *cspA* mRNA in *E. coli* undergoes post-transcriptional modification in response to environmental variations such as a temperature shift from 37°C to 10°C [37,38]. This RNA-dependent regulation of gene expression allows *E. coli* to rapidly adapt and respond to its environment. Further studies have shown that the *cspA* gene in *E. coli* produces a single-stranded nucleic acid-binding protein and an RNA chaperone. This protein is one of the most abundant proteins during early growth phase, and its expression is even higher during cold shock, accounting for 2% of the total proteins in the cell [39-41]. Since MsaB (CspA) is not directly involved in cold shock in *S. aureus*, it is not clear if it has maintained the same mechanism of regulation or functions as its homolog in *E. coli*. Based on our findings and those of Lioliou *et al*. [25], the MsaB transcript in *S. aureus* binds the anti-sense RNA (*msaR*), which contributes to its expression. *msaR* is detectable in the early and mid-exponential growth phases but not in the late exponential phase. The *msaB* transcript however is still present in late exponential growth phase albeit at a slightly lower level. It is not known at this point how this correlates with the production of the MsaB protein and what the significance of the differential expression of *msaR* is. It is also not known if the decrease in *msaR* in late exponential growth phase is due to increased degradation of the anti-sense RNA or lack of production of the transcript. Lioliou *et al*. [25] have shown that anti-sense RNA (*msaR*) binds to the 5′ UTR region of a long transcript of *cspA* (*msaB*) and prevents its processing by RNaseIII into a shorter transcript. The short transcript is presumably more stable than the long one and is translated more efficiently to produce the CspA (MsaB) protein. This processing might also be responsible to the abundance of the *msaB* transcript and suggests that it is the main product of the *msaABCR* operon. This also suggests that MsaB may be the main effector responsible for the functions we have shown such as biofilm development and regulation of virulence genes [21,22].

Our findings suggest that *msaC* produces a non-coding RNA. Our findings are supported by the absence of an MsaC protein from all proteomics studies in *S. aureus. MsaC* RNA is expressed in the 3′ end of the *msaB* transcript and its deletion leads to a significant reduction in *msaB* transcript [21] and mutant phenotypes that are similar to the deletion of the whole operon *msaABCR*. This suggests that *msaC* plays a regulatory role in the expression of MsaB. Interestingly, *msaC* is found both as an independent transcript and part of the large operon transcript (Figure 2). We were not able to detect an *msaC* promoter that is active under the conditions tested, which suggested that the smaller *msaC* transcript was the result of processing of the large operon transcript. The mechanism by which *msaC* regulates the expression of *msaB* is not clear and requires further studies.

The identification of the *msaABCR* operon will add insight into the complex network of virulence regulation in *S. aureus*. Despite the identification of numerous regulatory elements in *S. aureus*, it is still not clear how this organism achieves the coordinated expression of virulence factors in the host. Additionally, the strain-dependent differences observed in the pattern of regulation of virulence in *S. aureus* complicate this problem further [3]. Therefore, the addition of the *msaABCR* operon to the known repertoire of regulators used by *S. aureus* and studying its interactions with other regulators will improve our understanding of staphylococcal biology and the infectious process. This study was performed using a representative strain of the USA300 clonal lineage, whose hallmark phenotype is the high production of toxins, proteases, and phenol-soluble modulins [42,43]. It has been suggested that the unique regulation pattern of toxins in these stains is primarily responsible for their increased virulence and epidemic spread. The *msaABCR* operon positively regulates the *agr* operon and therefore may play an important role in the phenotype of this epidemic lineage of *S. aureus*. We plan to examine the contribution of the *msaABCR* operon to the fine-tuning of virulence expression via *agr* and other regulators.

## Conclusions

In this study, we identified a new operon, *msaABCR*, which regulates virulence and biofilm development in *S. aureus*. Two RNAs, *msaC* and *msaR*, regulate expression of this operon*.* The *msaC* gene was shown to be essential for the expression and function of the operon since its deletion resulted in a similar phenotype to deletion of the whole operon. Our findings indicated that the main transcript produced by the operon was *msaB,* which encodes the effector protein. We conclude that the pleiotropic effects observed by deletion of the *msaABCR* operon are probably mediated by its interactions with the global regulators *sarA, agr, and sigB*. Studies are underway to define the mechanism of regulation of the *msaABCR* operon and how it interacts with its target genes.

## Methods

### Bacterial strains and plasmids

*Staphylococcus aureus* strains (community-acquired MRSA strain USA300_LAC, restriction-deficient laboratory strain RN4220) and *E. coli* strain DH5α were used in this study. *S. aureus* strains were grown in tryptic soy broth (TSB) medium. Antibiotics (chloramphenicol (10 μg/ml), erythromycin (10 μg/ml), and kanamycin (50 μg/ml)) were used in TSB or TSA where needed. Similarly*, E. coli* strains were grown in LB broth with ampicillin (100 μg/ml) added where needed. Detailed information about the strains and plasmid constructs used in this study is listed in supplemental Additional file 4: Table S1.

### RNA isolation and real-time qPCR

Total RNA for the Smarter™ rapid amplification of cDNA ends (RACE) reaction was isolated from cells using a Qiagen RNeasy Maxi column (Qiagen, Valencia CA), as previously described in Sambanthamoorthy *et al.*[21]. Briefly, overnight cultures of *S. aureus* were diluted to an OD_600_ of 0.05 in TSB and incubated at 37°C with shaking (200 rpm) until they reached an OD_600_ of 1.5. The quality of total RNA was determined by Nanodrop spectrometer readings, as well as using a Bioanalyzer (Agilent). For real-time quantitative PCR (RT-qPCR) of the transcript, the total RNA was isolated from three different growth phases (early exponential, mid-exponential, and late-exponential), and RT-qPCR was performed as described previously [21]. The constitutively expressed *gyrase A* (*gyrA*) gene was used as an endogenous control gene and was included in all experiments. Analysis of expression of each gene was done based on at least three independent experiments. Two-fold or higher changes in gene expression were considered significant. All the primers used for RT-qPCR are listed in supplemental Additional file 5: Table S2.

### Analysis of RNA transcript by RACE

Analysis of RNA transcripts was carried out using the SMARTER™ RACE cDNA Amplification Kit as instructed in the user manual. The locations and sequences of gene-specific primers used for 3′ and 5′ RACE are shown in Figure 2 and Additional file 5: Table S2.

The 5′ RACE cDNA amplification was carried out using the random primer mix (N-15) provided in the kit. Alternatively, for confirmatory purposes, the 5′ RACE cDNA amplification was also performed using the poly (A)-tailed total RNA after poly (A) polymerization of the total RNA. The 5′-RACE-Ready cDNA was diluted to 100 μl and stored at -20°C until use. RACE was performed using universal primer mix, 5′ RACE primers, and the Advantage 2 Polymerase mix. Control experiments and all of the optimizations for the RACE reactions were performed as instructed in the manual. RACE-amplified product (5 μl) was resolved in a 1.2% gel to visualize the bands. The RACE products were gel purified and sequenced. The resulting sequence was used in a BLAST search on the NCBI website, and the 5′ end of the mRNA sequence was determined.

For the 3′ RACE reaction, the poly (A) tail was first added to the total RNA using the Poly (A) Polymerase kit. The 3′-RACE cDNA amplification was performed with 3′SMART CDS Primer A provided in the kit. The 3′-RACE-Ready cDNA was diluted up to 100 μl and stored at -20°C until use. RACE was performed using universal primer mix, 3′ RACE primers, and the Advantage 2 Polymerase mix. As above, control experiments and optimizations were performed, and the RACE products were visualized, gel purified, and sequenced. The resulting sequence was used in a BLAST search to determine the 3′ end of the mRNA sequence.

### Northern blot analysis

Total RNA for Northern blotting was harvested as described above. Cells were harvested at optical densities (*A*_600_) of 0.7, 1.5, and 4.0, which correspond to early-exponential, mid-exponential, and late-exponential, growth phases, respectively. Northern blots were performed using the DIG Northern starter kit, according to the manufacturer’s instructions (Roche Biochemicals, Mannheim, Germany). DIG-labeled riboprobes [200–300 bp] for *msaA, msaB, msaC*, and *msaR* were generated by transcription using the kit. The blotted membrane was prehybridized in 25 ml of Dig-Easy-Hyb buffer for 2 h at 50°C with rotation and hybridized in the same Dig-Easy-Hyb buffer containing 25 ng/ml DIG-labeled riboprobes overnight at 42°C. The hybridized membrane was first washed twice with 2× SSC and 0.1% SDS for 30 min at 37°C, followed by two 0.5× SSC and 0.1% SDS washes for 30 min at 50°C with rotation. After washing with 1× wash buffer (Roche) for 5 min, the membrane was incubated with blocking solution for 60 min and antibody solution (anti-DIG-alkaline phosphatase, 75 mU/ml) for 60 min at 37°C with rotation. The membrane was then equilibrated with 100 ml of detection buffer for 2–5 min and covered with 1 ml of the chemiluminescent substrate CDP-Star (Roche) for 10 min at room temperature, according to the manufacturer’s protocol. The membrane was immediately exposed to film for 3–30 min.

### Construction of promoter–*LuxAB* fusions and luciferase assays

The *E. coli*–staphylococcal shuttle vector pCN58, which contains the low-copy-number staphylococcal replicon cassette (pT181*copwt repC*) and a promoterless reporter gene, *luxAB* (encoding the luciferase from *Vibrio ficheri*) for transcriptional fusions [44], was used for the study of promoter activity of the individual ORFs (*msaA, msaB*, *msaC,* and *msaR*). The upstream 200–300-bp regions from individual genes were PCR amplified and cloned into the pCN58 vector. The recombinant vectors were first transformed into RN4220, followed by transduction into the USA300 LAC strains. To study the promoter–luciferase activity, overnight bacterial cultures were diluted 1:10 in TSB and further incubated for 3 h. Cells were then normalized to OD 0.05 and further incubated at 37°C with shaking (220 rpm). Bacterial cells (5 ml) were harvested at different optical densities (OD_600_ of 0.7, 1.5, and 4.0) representing early-exponential, mid- and late-exponential stages, respectively. The cells were washed once with 1× PBS and resuspended in 500 μl of 1× PBS. The cell suspension (500 μl) was mixed with 100 μl of 1% decanal (v/v) in 90% ethanol, and luminescence was measured immediately after mixing using a luminometer, based on a 10-s measurement in the integrated data mode. Luciferase activity was recorded as relative luminescence units (RLUs), and the specific luciferase activities were calculated by dividing the RLU values by the absorbance of the organism (RLU/OD_600_). The promoter-less version of the reporter gene plasmid (pCN58) was used as a control in reporter gene assays.

### Deletion of the *msaABCR* operon in the USA300 strain LAC and complementation

We used a previously described mutagenesis protocol to construct a nonpolar, in-frame deletion of the *msaABCR* operon in the *S. aureus* USA300 strain LAC [45]. Briefly, the flanking regions (~1 kb) of the *msaABCR* operon were amplified by PCR and ligated together at an introduced *Bam*HI restriction site. This PCR product was inserted into the temperature-sensitive plasmid pKOR1 using the Gateway BP Clonase Enzyme Mix (Invitrogen Inc.). The pKOR1–*msa* operon deletion construct was introduced into *S. aureus* USA300 strain LAC. The culture was grown in TSB in the presence of chloramphenicol (10 μg/ml) at the permissive temperature of 30°C. Cells were plated on TSA containing chloramphenicol at 43°C, a non-permissive temperature for pKOR1 replication. Colonies were picked and allowed to grow in TSB at the permissive temperature and then plated on TSA containing 100 ng/ml of anhydrotetracycline, which induces antisense *secY* RNA expression and promotes loss of plasmid. Two rounds of temperature shifts were necessary to isolate the deletion mutant. Deletion of *msaABCR in* LAC was verified by end-point and real-time PCR, and functional assays were performed as described previously [21]. To complement the *msaABCR* deletion mutation, a 1788-bp fragment of the *msaABCR* operon gene with complete 5′ and 3′ untranslated regions was amplified and ligated to pCN34 (NARSA), a low-copy-number, Gram-positive shuttle vector. The complement plasmid construct was introduced into strain RN4220 by electrophoresis and then transduced into the *msaABCR* deletion mutant. The *msaABCR* operon gene in the complemented strain was under the control of its native promoter. We sequenced the *agr* operon of the *msaABCR* mutant and compared it with the parent strain to ensure that it had not spontaneously mutated during construction of the *msaABCR* operon deletion mutant.

### Mutagenesis study

We used an overlap extension PCR cloning technique to generate a frame shift mutation in the *msaC* ORF as described in Bryksin *et al*. [46]. The upper 1120-bp fragment of the *msaABCR* operon was PCR amplified using primer set fsmut-*msa* F1 and fsmut-*msa* R1, and the lower 650-bp fragment of the *msaABCR* operon region was amplified using primer set fsmut-*msa* F2 and fsmut-*msa* R2. Primers fsmut-*msa* R1 and fsmut-*msa* F2 overlap such that a deletion of one nucleotide was introduced causing a frameshift mutation in *msaC*. Both of the PCR fragments were PCR purified using the Promega DNA cleanup kit, and then 50 ng of each of the fragments were used in the PCR ligation, which contained all of the components of the PCR mix except the terminal primers. The normal PCR cycle was carried out for 15 cycles, then the terminal primers (fsmut-*msa* F1 and fsmut-*msa* R2) were added to the reaction and an 20 additional cycles were performed. The final amplified PCR product was ligated to the pCN34 low copy number plasmid vector and transduced into the *msaABCR* operon deletion mutant.

### Pigmentation assay

A pigmentation assay was performed on cells harvested from overnight cultures, as described by Morikawa *et al*. [47]. Briefly, 1 ml of the cells were harvested and washed twice with water. They were then suspended in 1 ml of methanol and heated at 55°C for 3–5 min with occasional vortexing. The cells were removed by centrifugation at 15,000 × *g* for 1 min, and the absorbance of the supernatant was measured at 465 nm with water as a blank. Mean values from a minimum of three independent experiments, each performed in triplicate, were recorded.

### Protease assay

Protease activity was measured from the supernatants of 4-h and overnight cultures as described by Sambanthamoorthy *et al*. [21]. Briefly, 300 μl of the culture supernatant was mixed with 800 μl of 3 mg azocasein ml^-1^ in Tris-buffered saline (pH 7.5) and incubated overnight at 37°C. Undegraded azocasein was precipitated by adding 400 μl of 50% (w/v) trichloroacetic acid, removed by centrifugation and the amount of acid-soluble azocasein was determined by measuring the *A*_340_. Mean values from a minimum of three independent experiments, each performed in triplicate, were recorded.

### Biofilm assays

The microtiter biofilm assay was performed as described in Sambanthamoorthy *et al*. [22] with slight modification. In brief, overnight cultures of cells, including wild type, mutant, and the complemented strain of USA300 LAC were diluted 1:100 times in TSB supplemented with 3% NaCl and 0.25% glucose and inoculated in microtiter plates pre-coated with 20% human plasma. Cultures were incubated for 24 or 48 h with shaking at 150 rpm. The adherent biofilm was quantitated at 615 nm after washing and staining with crystal violet and elution with 5% acetic acid. Mean values from a minimum of three independent experiments, each performed in triplicate, were recorded.

### Ethics statement

This research did not involve human subjects, human material, or human data.

## Abbreviations

ORF: Open reading frame; RACE: Rapid amplification of cDNA ends; RLU: Relative luminescence unit; RT-qPCR: Real-time quantitative PCR.

## Competing interests

The authors declare to have no competing interests.

## Authors’ contributions

GSS and MOE designed and performed the experiments. GSS and MOE wrote the manuscript. All authors read and approved the final manuscript.

## Supplementary Material

Additional file 1: Figure S1 Absolute quantification of individual ORFs in the *msaABCR* operon. Real-time quantitative PCR was used to compare the expression level of three genes in the *msaABCR* operon in three growth phases (early exponential, mid exponential, and late exponential). These results confirmed the findings from Northern blot analysis showing that despite co-expression of all genes, the *msaB* transcript was the most abundant. Results were obtained from three independent experiments. Values represented the mean ± S.E. Click here for file

Additional file 2: Figure S2 Activity of the *msaABCR* promoter. Activity of the primary promoter was measured in the *msaC* and *msaABCR* deletion mutants. Luciferase activity was measured at three planktonic growth phases (early, mid, and late exponential) and biofilm growth. The vector pCN58, containing *luxAB* without a promoter, was used as a negative control (not shown). Results represent the means of three independent experiments, where each measurement was done in triplicate. Values represent the mean ± S.E. Click here for file

Additional file 3: Figure S3 Predicted structure of *msaC* RNA and the putative protein sequence. A frame shift mutation (deletion of U) was introduced into the *msaC* gene of the *msaABCR* operon. The predicted structure of the *msaC* RNA in the wild type **(A)** and mutant **(B)** strain showed no significant difference in secondary structure. The predicted protein sequence of the wild type **(C)** and mutant **(D)** strain showed the introduction of several stop codons. Click here for file

Additional file 4: Table S1 Strains and plasmids used in this study. Click here for file

Additional file 5: Table S2 Primers used in this study. Click here for file
