# Supplementary figures and images for: Identification and characterization of an operon, msaABCR, that controls virulence and biofilm development in Staphylococcus aureus
Source: BMC Microbiol. 2014 Jun 11;14:154. doi: 10.1186/1471-2180-14-154 (PMC4229872; doi:10.1186/1471-2180-14-154)

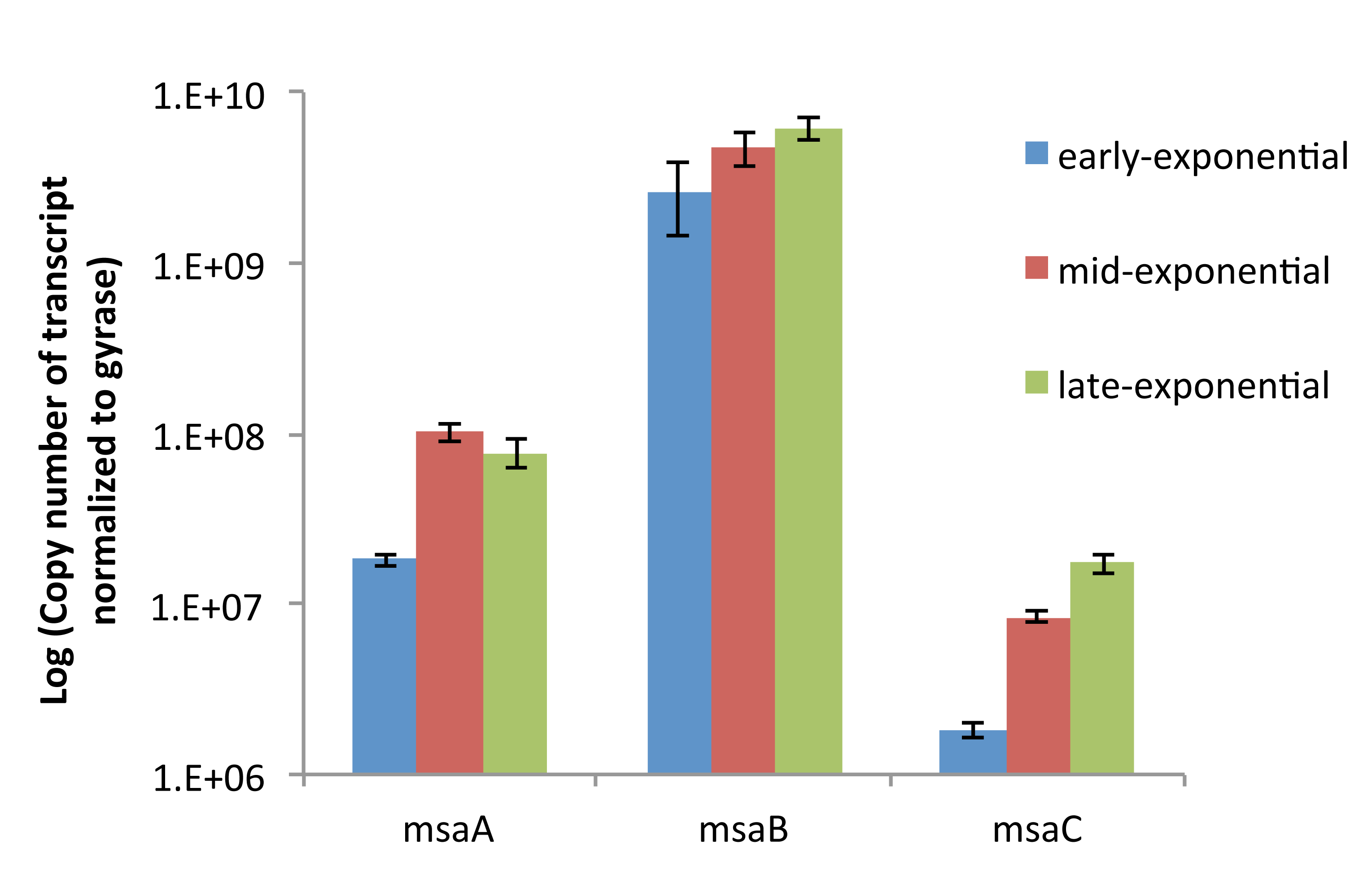

Supplement: Additional file 1: Figure S1 — Absolute quantification of individual ORFs in the msaABCR operon. Real-time quantitative PCR was used to compare the expression level of three genes in the msaABCR operon in three growth phases (early exponential, mid exponential, and late exponential). These results confirmed the findings from Northern blot analysis showing that despite co-expression of all genes, the msaB transcript was the most abundant. Results were obtained from three independent experiments. Values represented the mean ± S.E. [file 1471-2180-14-154-S1.tif]

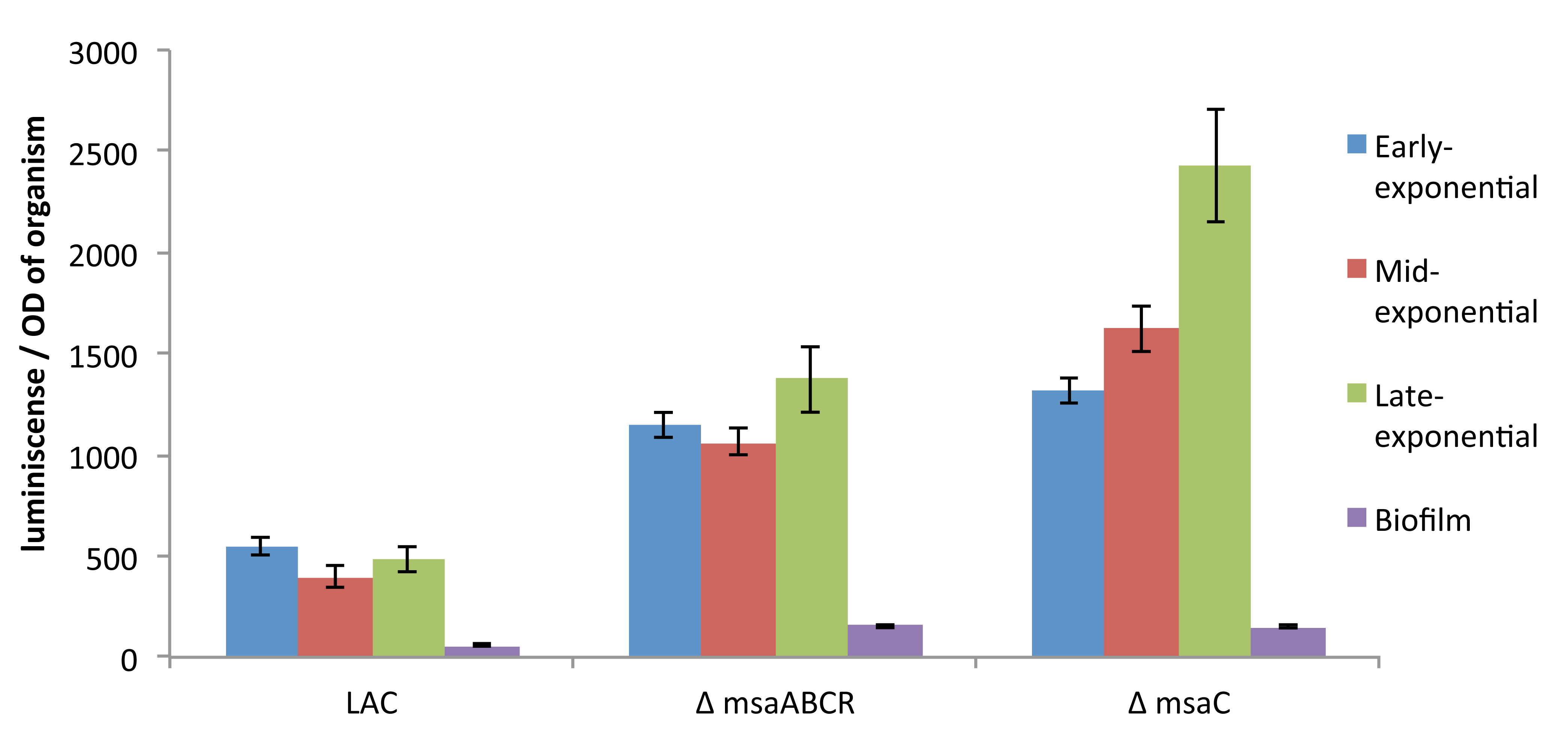

Supplement: Additional file 2: Figure S2 — Activity of the msaABCR promoter. Activity of the primary promoter was measured in the msaC and msaABCR deletion mutants. Luciferase activity was measured at three planktonic growth phases (early, mid, and late exponential) and biofilm growth. The vector pCN58, containing luxAB without a promoter, was used as a negative control (not shown). Results represent the means of three independent experiments, where each measurement was done in triplicate. Values represent the mean ± S.E. [file 1471-2180-14-154-S2.tif]

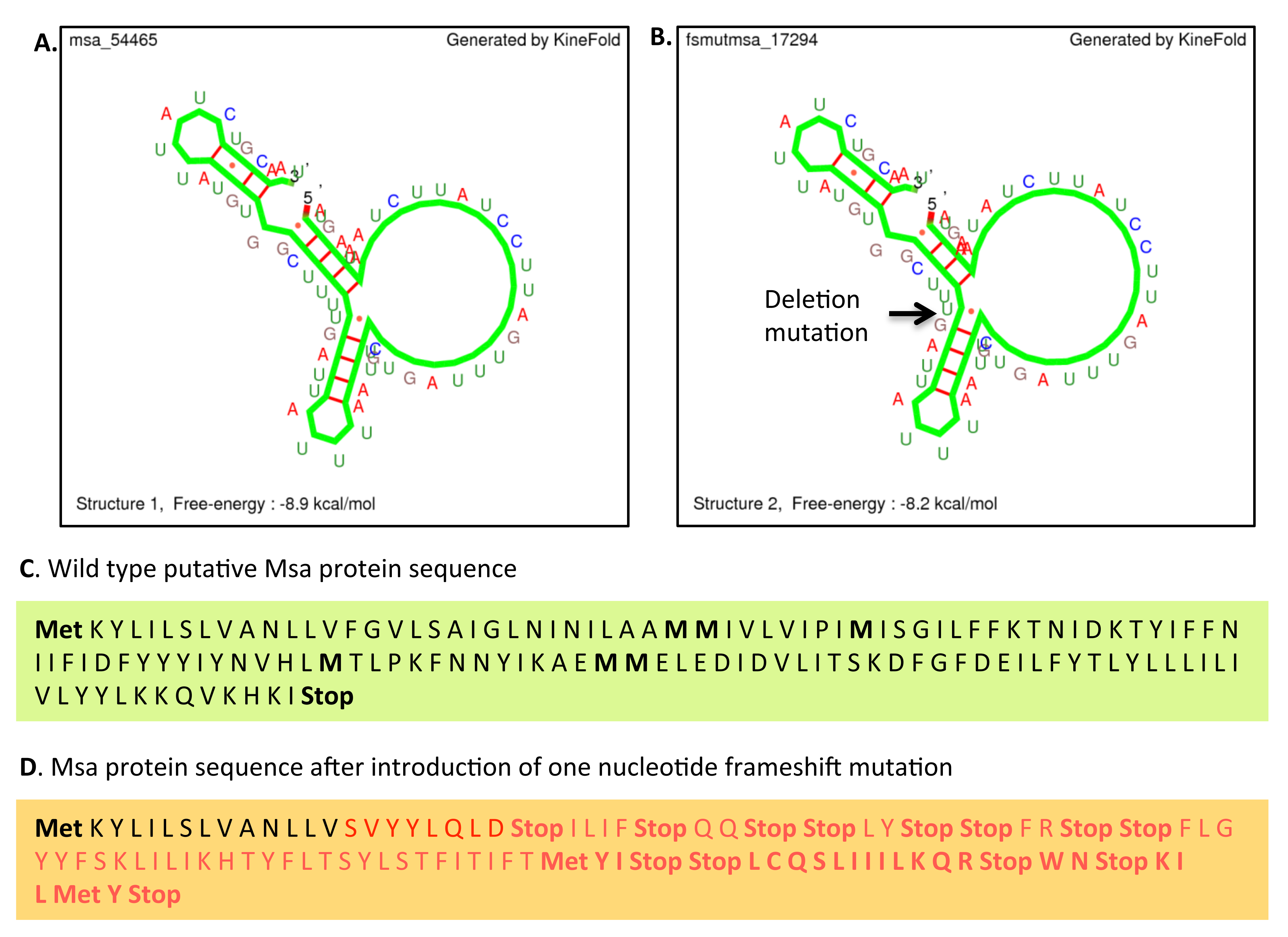

Supplement: Additional file 3: Figure S3 — Predicted structure of msaC RNA and the putative protein sequence. A frame shift mutation (deletion of U) was introduced into the msaC gene of the msaABCR operon. The predicted structure of the msaC RNA in the wild type (A) and mutant (B) strain showed no significant difference in secondary structure. The predicted protein sequence of the wild type (C) and mutant (D) strain showed the introduction of several stop codons. [file 1471-2180-14-154-S3.tif]
