# Supplementary material for: Identification and characterization of an operon, msaABCR, that controls virulence and biofilm development in Staphylococcus aureus
Source: BMC Microbiol. 2014 Jun 11;14:154. doi: 10.1186/1471-2180-14-154 (PMC4229872; doi:10.1186/1471-2180-14-154)
Supplement: Additional file 4: Table S1 — Strains and plasmids used in this study. [file 1471-2180-14-154-S4.docx]

**Table S1. Strains and plasmids used in this study**

| **Plasmids** | | |
| --- | --- | --- |
| **Plasmid** | **Relevant characteristics** | **Source** |
| pKOR1 | E. coli-S. aureus shuttle vector, ori(Ts) inducible, secY antisense counterselection, Amp^r^ Cm^r^ | Dr. Taeok Bae |
| pMOE 399 | pKOR1: Δ *msa* operon deletion construct | This study |
| pCN34 | pT181-based low copy number E. coli-Staphylococcal shuttle vector | NARSA |
| pMOE 402 | pCN34-*msaC* gene :: *msaC* gene complement | This study |
| pMOE 403 | pCN34-*msaABCR* operon :: *msaABCR* operon complement | This study |
| pCN58 | pT181-based *E. coli*-staphylococcal shuttle vector that contains promoterless LuxAB as a reporter gene for promoter-gene fusion. | NARSA |
| pMOE 481 | pCN58-Promoter *^msaA^* | This study |
| pMOE 482 | pCN58-Promoter *^msaB^* | This study |
| pMOE 483 | pCN58-Promoter *^msaC^* | This study |
| pMOE 498 | pCN58-Promoter *^msaR^* | This study |
| pMOE 501 | pCN58-Promoter *^sarAP1^* | This study |
| pMOE 555 | pCN34-*msaABCR*_fsmut *msaC* |  |
| **Strains** | | |
| **Strain** | **Relevant characteristics** | **Reference or source** |
| RN4220 | Restriction deficient mutant of 8325-4 | NARSA |
| LAC | CA-MRSA USA300 strains | Dr. Lindsey Shaw |
| MOE 383 | LAC :: Δ *msaC* deletion mutant | This study |
| MOE 401 | LAC :: Δ *msaABCR* operon deletion mutant | This study |
| MOE 392 | MOE 383 :: pMOE 402 :: *msaC* gene complement into *msaC* deletion mutant | This study |
| MOE 393 | MOE 383 :: pMOE 403 :: *msaABCR* complement into *msaC* deletion mutant | This study |
| MOE 404 | MOE 401 :: pMOE 403 :: *msaABCR* complement into *msaABCR* deletion mutant | This study |
| MOE 555 | MOE 401 :: pMOE 555 :: *msaABCR*_Fsmut *msaC* complement into *msaABCR* deletion mutant | This study |
| MOE 413 | MOE 383/pCN34-vector control | This study |
| MOE 414 | MOE 401/pCN34-vector control | This study |
| MOE 481 | LAC :: pCN58-Promoter *^msaA^* | This study |
| MOE 482 | LAC :: pCN58-Promoter *^msaB^* | This study |
| MOE 483 | LAC :: pCN58-Promoter *^msaC^* | This study |
| MOE 484 | MOE 383/ pCN58-Promoter *^msaA^* | This study |
| MOE 485 | MOE 383/ pCN58-Promoter *^msaB^* | This study |
| MOE 486 | MOE 383/ pCN58-Promoter *^msaC^* | This study |
| MOE 487 | MOE 401/ pCN58-Promoter *^msaA^* | This study |
| MOE 488 | MOE 401/ pCN58-Promoter *^msaB^* | This study |
| MOE 489 | MOE 401/ pCN58-Promoter *^msaC^* | This study |
| MOE 495 | LAC :: pCN58-vector control | This study |
| MOE 496 | MOE 383/ pCN58 vector control | This study |
| MOE 497 | MOE 401/ pCN58 vector control | This study |
| MOE 498 | MOE / pCN58-Promoter *^msaR^* | This study |
| MOE 499 | MOE 383/ pCN58-Promoter *^msaR^* | This study |
| MOE 500 | MOE 401/ pCN58-Promoter *^msaR^* | This study |
| MOE 501 | MOE /pCN58-Promoter *^sarAP1^* | This study |
| MOE 502 | MOE 383/pCN58-Promoter *^sarAP1^* | This study |
| MOE 503 | MOE 401/pCN58-Promoter *^sarAP1^* | This study |
