# Supplementary material for: Identification and characterization of an operon, msaABCR, that controls virulence and biofilm development in Staphylococcus aureus
Source: BMC Microbiol. 2014 Jun 11;14:154. doi: 10.1186/1471-2180-14-154 (PMC4229872; doi:10.1186/1471-2180-14-154)
Supplement: Additional file 5: Table S2 — Primers used in this study. [file 1471-2180-14-154-S5.docx]

**Table S2. Primers used in this study**

| **Primers used in RACE reactions** | |
| --- | --- |
| 5 RACE *msaC* | CCCAGAAATCATTATCGGAATCACTAATACAATCA |
| 5 RACE *msaC* 1 | TACCACACTTTAAACAAGCATCATGAAATTAG |
| 3 RACE *msaC* | TAGTTGCTAATTTATTAGTTTTCGGTGTATTAT |
| 5 RACE *msaB* | TATAGTTTAACAACGTTTGCAGCTTGTGGAC |
| 3 RACE *msaB* | ACGCTGAAAAAGGATTCGGCTTTATCGAAGTTGAA |
| 5 RACE *msaA* | ATAAAATATTTGTAAATCCTCTTCACAATCTTCG |
| 3 RACE *msaA* | CAGTTACCAAAATTAGAGCCGATTATGAGGGATGG |
| 5 RACE *msaR* | AGCAGATGATTATTCCATATTGCAAAGAATATTTGAGTGA |
| 3 RACE *msaR* | ACTGTACCTTGTTTCATAATCTGAAACCTCCAAGACTA |
| **Primers used in Northern blot analysis** | |
| NB *msaC* R | TAATACGACTCACTATAGGGCAACAAATATAATGTATAAAACAGAATTTCATC |
| NB *msaC* F | GATTCCGATAATGATTTCTGGGATACTATTTT |
| NB *msaB* R | TAATACGACTCACTATAGGGTATAGTTTAACAACGTTTGCAGCTTGTGGAC |
| NB *msaB* F | TTAAATGGTTTAACGCTGAAAAAGGATTCG |
| NB *msaA* R | TAATACGACTCACTATAGGGATAAAATATTTGTAAATCCTCTTCACAATCTTCG |
| NB *msaA* F | GTGGACAGTTACCAAAATTAGAGCCGATTAT |
| NB *msaR* F | AATTAACCCTCACTAAAGGGAGCAGATGATTATTCCATATTGCAAAGAAT |
| NB *msaR* R | AATCTGAAACCTCCAAGACTACCCTTCATTCA |
| **Primers used in RT-qPCR** | |
| RT *msaC* F | CCAGAAATCATTATCGGAATCACTA |
| RT *msaC* R | TTAGTTTTCGGTGTATTATCTGCAA |
| RT *msaB* F | TTTATCGAAGTTGAAGGAGAAAATG |
| RT *msaB* R | ACTCAACAGCTTGACCTTCTTCTAA |
| RT *msaA* F | TCGATAACTATGTCACAGGCAAATA |
| RT *msaA* R | TTGTAAATCCTCTTCACAATCTTCG |
| RT *agrA* F | TTTGTCGTCAATCGCCATAA |
| RT *agrA* R | TTTAACGTTTCTCACCGATGC |
| RT *sarA* F | TTTGCTTCAGTGATTCGTTTATTTACTC |
| RT *sarA* R | GTAATGAGCATGATGAAAGAACTGTATT |
| RT *sigB* F | GAAGCTAAGTCTATCTCTTTATCGTGAA |
| RT *sigB* R | CAAGAAATCGTTAAAGGCTTTGGTTATA |
| RT *gyrA* F | GCTCGTTCGTGACAAGAAAA |
| RT *gyrA* R | TTTGCATCCTTACGCACATC |
| **Primers used for operon deletion and complementation** | |
| *msa*ABCR upstr attB1 F | GGGGACAAGTTTGTACAAAAAAGCAGGCTGCTTTAAATCAGCGATTAATGTTCGTTTG |
| *msa* operon upstr R | ATGACTGGATCCTATTAAAGACCCCTTCCATACTTCAAAAAC |
| *msa* operon dnstr F | ATGACTGGATCCTTTCATGATGCTTGTTTAAAGTGTGGTAT |
| *msa* operon dnstr attB2 R | GGGGACCACTTTGTACAAGAAAGCTGGGTAGTTTGGATTATCAATTCAATATGGCTTAGC |
| *msa* operon F | TAAAATATCTGGATCCGACGCCTT |
| *msa* operon R | ATAAAGCTGCAGTCGTTAAGACAAC |
| *msaC* compl F | ACCGCGGTACCCAAGCTGCAAACGTTGTTAAACTA |
| *msaC* compl R | AAGCACGTGAATTCATAAAGCGACAATCGTTAA |
| *msaABCR* compl F | GGGGGATCCTTTTACCACCTCATAATGTTAT |
| *msaABCR* compl R | CCCGAATTCAAATAAACAAAGTAATCCCCGA |
| **Primers used to measure promoter activity** | |
| Pro-*msaA* F | CAATGCGGATCCTAAAATATCTAAATCCGACGCCT |
| Pro-*msaA* R | TCTGGCCAGGTACCGAATAACCACCATCCCTCATA |
| Pro-*msaB* F | TGCGAAGATGGATCCGAGGATTTACAAATATTTTA |
| Pro-*msaB* R | ACGTCATTGGTACCTTCAACTTCGATAAAGCC |
| Pro-*msaC* F | AGGTCAAGGATCCGAGTTTGAAGTAGTTGAAGGCG |
| Pro-*msaC* R | CATTATCGGTACCACTAATACAATCATCATTGCTG |
| Pro-*msaR* F | AATAAACAAAGGGATCCCCGAAGCACAGAAAATTA |
| Pro-*msaR* R | ACGCTGAAAAAGGTACCGGCTTTATCGAAGTTGAA |
| Pro-*sarAP1*-F | ACTAGGGATCCCTGATATTTTTGACTAAACCAAATGC |
| Pro-*sarAP1*-R | ACTAGGGTACCGATGCATCTTGCTCGATACATTTG |
| **Primers used to construct frameshift mutant** | |
| fsmut-*msaC* F1 | ACTTGTAAATGGATCCGTCTCATTTTTACCACCTCA |
| fsmut-*msaC* R1 | GCAGATAATACACCGAAACTAATAAATTAGCAACT |
| fsmut-*msaC* F2 | AGTTGCTAAATTATTACTTTCGGTGTATTATCTGC |
| fsmut-*msaC* R2 | AGTTATAAAGGTACCATCGTTAAGACAACTCATTA |
